# A Comparative Study of Friction and Wear Processes of Model Metallic Biomaterials Including Registration of Friction-Induced Temperature Response of a Tribological Pair

**DOI:** 10.3390/ma12244163

**Published:** 2019-12-11

**Authors:** Magdalena Łępicka, Artur Ciszewski, Karol Golak, Małgorzata Grądzka-Dahlke

**Affiliations:** 1 Department of Materials and Production Engineering, Faculty of Mechanical Engineering, Bialystok University of Technology, 15-351 Bialystok, Poland; k.golak@pb.edu.pl (K.G.); m.dahlke@pb.edu.pl (M.G.-D.); 2 Faculty of Mechanical Engineering, Bialystok University of Technology, 15-351 Bialystok, Poland

**Keywords:** wear, biomedical alloys, temperature effect

## Abstract

Nowadays, metallic alloys are extensively used in wear-related biomedical applications. However, it was shown that one of the factors which may contribute to the premature implant failure is the temperature effect caused by the sliding action between the bearing surfaces. Nevertheless, there are not many papers where the wear-related temperature phenomena of biomedical alloys are discussed. Thus, in our paper, we present findings from the tribological tests of the model metallic biomaterials—316L steel, CoCrMo alloy and Ti gr. 2. In our study, the temperature alterations induced by the wear action of the examined materials were analyzed. According to the findings, the temperature response of the biomedical alloys is tribological pair dependent. While the mass loss of the tribological pair 316L–316L steel was the slightest, at the same time the temperature increase was the greatest. Based on the presented findings, further analyses in friction-induced temperature response of biomedical alloys is recommended.

## 1. Introduction

In the recent decades, an extensive research in the field of biomaterials for artificial joint applications was done. The extended life expectancy, the lifestyle based on maintaining health and wellbeing through physical exercises, as well as the need for maintaining a comfortable life are the cause for the constant development of modern biomaterials. An exceptional awareness is given especially to the long-term durability and reliability of orthopedic implants.

The main causes for premature failure of the orthopedic implants are the insufficient operational properties of the materials used in the artificial joints applications. Both tribological wear and the resulting wear debris are the important causes not only for aseptic loosening of the orthopedic implants but also for the onset of local inflammations [1]. Due to this, solutions which maximize the patients’ safety are still sought.

In general, the popular metallic materials for orthopedic implants are: 316L stainless steel, CoCrMo alloys, as well as Ti and its alloys [2]. In many cases, those materials are used in wear-related applications. For example, though 316L steel is virtually not used anymore in primary implants, in veterinary applications it is a first-choice material when a revision surgery of a hip implant is needed [3].

In clinical practice, both the metal-on-metal (MoM) as well as the metal-on-polyethylene (MoP) bearing surfaces are used. Though, in general, the MoM implants are characterized by a smaller volumetric wear than the MoP type endoprostheses [4], the legitimacy of use of the MoM joint endoprostheses is now being questioned, for example, by the U.S. Food and Drug Administration [5]. It has been shown that in MoM hip implants, metallic particles are released to the body due to sliding action of the mating surfaces [6]. The particles can be micro- or nanosized [7], which makes it possible for them to enter not only the surrounding tissues but also the bloodstream. Moreover, it has been shown that the tribocorrosion processes, which are induced by the physiological fluids, can enhance the severity of tribological wear in implant materials [8]. Nowadays, the measurements of metals (particularly Cr and Co) in the blood are used as an evidence of the artificial joint wear [9]. An increase up to 100-fold in cobalt and chromium levels, from preoperative to postoperative values, has been demonstrated in multiple implant systems [9].

To improve the wear and corrosion performance of implant alloys, numerous coatings, e.g., titanium nitride (TiN) and diamond-like-carbon (DLC) [10], as well as the thermochemical treatments [11], are used. Nevertheless, it has been shown that in the long-term in vivo usage, the protective films can be worn out of the implant surface [12]. The release of the particles of hard coatings to the bearing surfaces may result in the local increase of surface roughness, as well as enhance tribological wear of the mating parts due to three-body abrasive action [12]. Due to that, it can be stated that up to this date, no optimal biomedical metallic material for wear-related purposes has been found.

In many cases, the most commonly used methods for wear testing of the metallic biomaterials are not particularly aimed on reflecting the actual working conditions of an artificial joint. For example, for tribological testing purposes, the ball-on-disc [13,14,15], pin-on-disc [13,15] and block-on-disc [16] configuration might be used. Moreover, the lubricating conditions [14,17,18,19,20], the applied load [13,15,20], the sliding velocity [13,21] as well as the temperature [16,19], may differ. Because of that, it is challenging to compare between the authors results from the tribological tests. Studies aimed at determining the reliability of the newly proposed, original material solutions, should reflect the operating conditions of the artificial joint as closely as possible; that is, the contact geometry, pressures, sliding speeds, temperature, and lubricating fluids are selected to mimic the service conditions of a natural joint. However, in comparative tests of various materials, some simplifications can be done in order to eliminate additional unknowns, e.g., the actual contact surface when the spherical head and acetabulum are in contact while a given gap in maintained between them.

Therefore, further analyses in the wear behavior of biomedical metallic materials are needed. As presented in the literature, the implant materials should not be analyzed in terms of release of the wear debris only. According to Bergmann et al. [22], one of the factors which may contribute to the premature implant failure is the temperature effect caused by the sliding action between the bearing surfaces. Thus, in our paper, we present a comparison between the model metallic biomaterials, 316L steel, CoCrMo alloy, and pure titanium. During the wear test, the temperature alterations caused by friction were recorded. To our knowledge, there are not many reports from the wear tests of biomedical alloys, where the temperature measurements were included.

## 2. Materials and Methods

### 2.1. Materials

For the wear test purposes, three metallic materials were chosen: 316L stainless steel, Co28Cr6Mo cobalt alloy as well as Ti gr. 2. All those materials are currently considered the popular metallic materials for biomedical applications. From those materials, pins of diameter equal to 8 mm and length L = 12 mm were fabricated. Surface treatment that was applied on all the samples was analogous to the one that is used for biomedical implants. Due to this, all samples were ground and, after that, polished. The average roughness Ra of the working surface, which was calculated from 5 measurements, as well as the microhardness of the considered samples are presented in Table 1.

### 2.2. Methods

**Figure 1:**
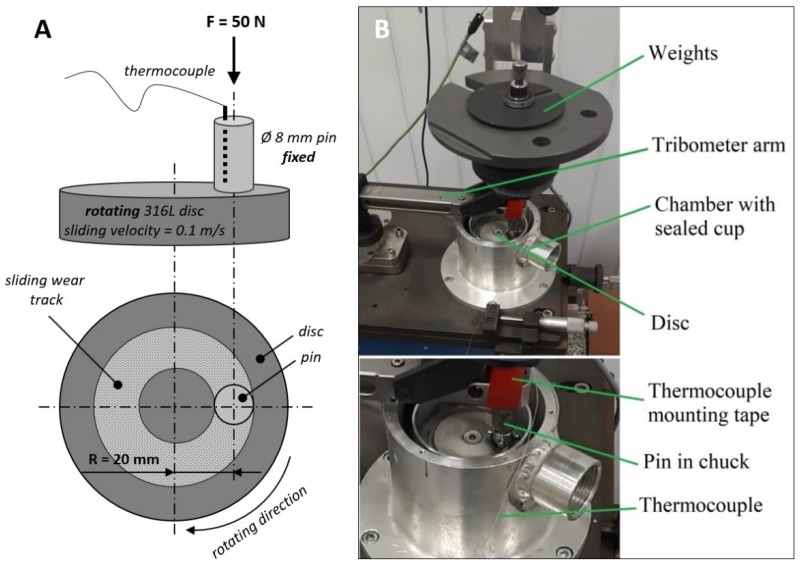
The pin-on-disc wear test system used in the study: (**A**) schematic representation of the tribological pair, (**B**) the actual setup of the tribometer.

The sliding wear tests were conducted on a T-11 tribometer (Institute for Sustainable Technologies, Radom, Poland) in a pin-on-disc configuration (Figure 1A), under the load of 50 N. As pins, 316L, CoCrMo and Ti gr 2. were used (Table 1), while the counter samples, the Ø50 × 10 mm discs, were fabricated from 316L steel (average Ra = 0.150 µm). The wear track radius equaled 20 mm, while the sliding velocity was 0.1 m/s. The sliding velocity of 0.1 m/s was selected considering one of the applications of metallic biomaterials. As presented by Damm et al. [26], the sliding speed of mating parts of an implanted hip endoprosthesis during its natural flexion and extension may differ between the individual patients (Table 2). According to the literature data presented in Table 2, the average in vivo sliding speed of a hip implant measured during walking equals 0.03 m/s during extension and 0.06 m/s during flexion. However, due to suppressing action of the disc during sliding, the rotating speed of the counter sample had to be set to a higher value than presented in Table 2. Application of up to 3 times greater sliding speed than it would result from data presented in Table 2 was implied by the technological properties of the testing device. Nevertheless, the set sliding speed should not significantly affect the friction conditions and the mechanical wear of contacting materials. The actual test setup is presented in Figure 1B. In tribological tests, the total sliding time equaled 7200 s, what corresponds to 720 m of wear distance. The laboratory temperature was 21 °C. In each subset, three samples were examined. The morphology of the resultant wear tracks on both pins and counter samples was examined using an electron scanning microscope (SEM, Hitachi S-3000N, Hitachi, Tokio, Japan) equipped with an energy dispersive X-ray (EDX) sensor.

During the test, changes of friction force in time were registered. Using the friction force, the coefficient of friction (COF) was calculated with the following formula:

µ = T/F(1)

where T is the friction force, and F is the applied force (F = 50 N). Moreover, in order to determine wear of all tested tribological pairs, the wear-related changes in weight of both pin and disc were measured. Before and after the wear tests, all the samples were weighed on a laboratory scale with an accuracy of 10^−4^ g.

What is more, the friction-induced temperature alterations were monitored by registering the pin temperature in the proximity to the wear zone. For this purpose, in top part of the pins, solitary blind holes that were finishing 2 mm from the working surface of the pins were made with an electrical discharge machining (EDM)technology. In the blind holes, OMEGA type K thermocouples (Omega Engineering Inc., Norwalk, CT, USA) with sensing temperature range from −200 to 1250 °C and standard limits of error equal to 0.75% were installed. The temperature alterations of the pin were recorded with a frequency of 1 Hz.

The metallic implant materials do not work in dry sliding conditions. Therefore, for our comparative study purposes, the simplest solution, a 0.9% NaCl physiological fluid, which is often used in biotribological studies [18,27,28], was chosen. To ensure the presence of the fluid in the friction zone, the rotating 316L disc was fixed in an aluminum cup. The cup worked as a container, where 25 mL of a fresh saline solution was put for the test purposes. After each experiment, the wear test setup was thoroughly cleaned, the wear particles collected, as well as new portion of saline solution was poured in the aluminum container.

## 3. Results and Discussion

**Figure 2:**
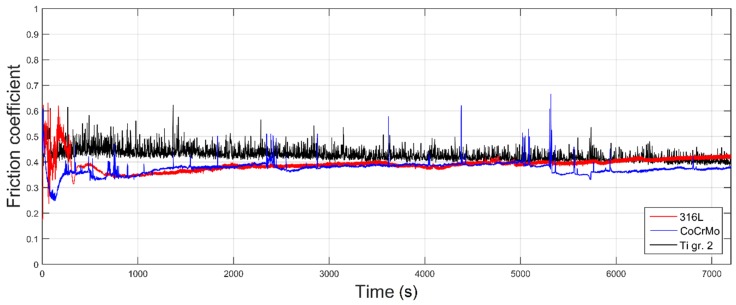
Variations of the coefficient of friction registered during the wear test; red, 316L stainless steel; blue, CoCrMo; black, Ti gr. 2. The test was conducted under the load of 50 N, in an 0.9% NaCl solution. Total sliding time: 7200 s, corresponding to 720 m of sliding.

**Figure 3:**
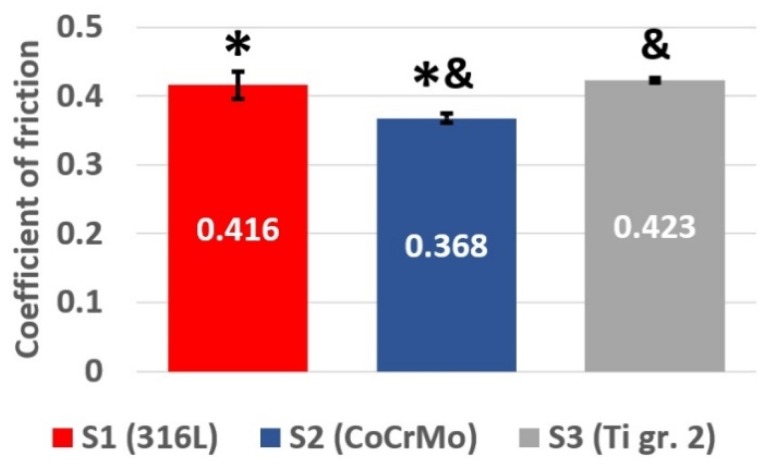
Comparison of the average COFs measured for all the tribological pairs tested; red, 316L stainless steel; blue, CoCrMo; black, Ti gr. 2. The average values were calculated from the stable friction state. Marks *^&^ are used to denote the groups between which the statistically significant difference exists (one-way ANOVA, α = 0.05; post-hoc Tukey HSD test, α = 0.05).

Figure 2 presents the fluctuations of the coefficient of friction (COF) registered for 316L stainless steel, CoCrMo cobalt alloy and Ti gr. 2 during 7200 s of friction. As can be seen, in case of all tested materials, the COF plot can be divided in two sections: the abrupt running-in state and the stabilized friction. In Figure 3, the average values of COF are presented for all analyzed material groups. The depicted values of COF were calculated for the stabilized friction state. As presented in Figure 3, there is statistically significant difference in terms of value of the average COF in the stabilized state between the tested groups (one-way ANOVA, α = 0.05; post-hoc Tukey HSD test, α = 0.05). This implies that differences in friction behavior will be observed between the analyzed sets of the samples. The coefficient of friction of the tribological pair 316L vs. CoCrMo is the lowest one amongst all tested specimens.

**Figure 4:**
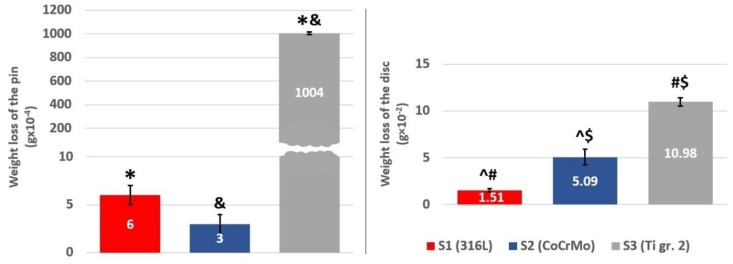
The average relative mass loss of the examined samples; mean ± SD; red, 316L stainless steel; blue, CoCrMo; black, Ti gr. 2. Marks *^&^#$^ are used to denote the groups between which the statistically significant difference exists (one-way ANOVA, α = 0.05; post-hoc Tukey HSD test, α = 0.05).

Nevertheless, though there is a statistically significant difference in average COFs, for all tested materials, the values of COFs are quite similar (Figure 3). However, this observation is not reflected in the weight loss and the resultant wear rate of the analyzed sets of samples (Figure 4). An attempt to justify the findings from the weight loss measurements could be made by comparing the microhardness of the pin materials (Table 1). However, comparison in terms of microhardness of the materials does not provide the answer for the question why is wear of Ti gr. 2 three magnitudes of order greater than that of the other pins. Therefore, the temperature alterations in the proximity to the friction zone were registered and analyzed for all tested materials.

**Figure 5:**
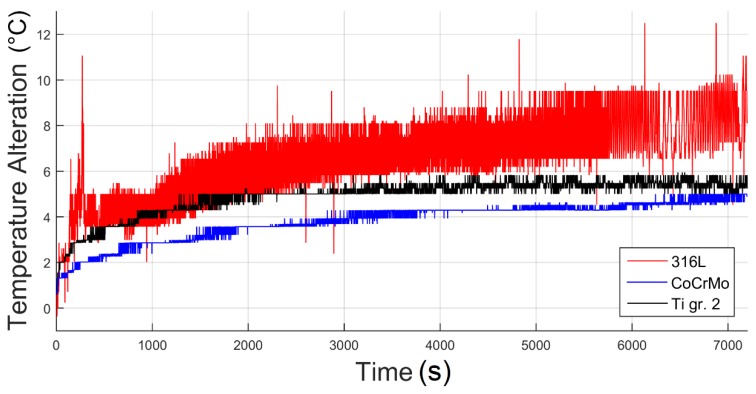
Variations of the temperature registered during the wear test; red, 316L stainless steel; blue, CoCrMo; black, Ti gr. 2. The test was conducted under the load of 50 N, in an 0.9% NaCl solution. Total sliding time: 7200 s, which corresponds to 720 m of sliding.

The observed differences in terms of COF and weight loss between the analyzed samples resulted in differences in the thermal response of the materials (Figure 5). According to O’Donell et al. [29], abrupt changes in friction can be observed; when as a result of increase of the sliding speed in the tribological contact, frictional heating and temperature increase at the interface of the sliding material can be seen.

As it could have been suspected, at the beginning of friction, the temperature measured in the proximity to the friction zone increases (Figure 5). Nevertheless, some differences between the analyzed tribological pairs can be observed. In case of Ti gr. 2 and CoCrMo, in the course of time the temperature alterations are very similar. However, the difference in terms of temperature response of those materials can be seen in values of the registered temperatures. Nevertheless, this variation can be caused by the different heat conductivity of both Ti gr. 2 and CoCrMo. On the other hand, for 316L steel, the increase in temperature is substantially greater (Figure 5). Moreover, the temperature vs. time plot is characterized by much greater oscillations.

When analyzing data plotted in Figure 5, it can be seen that the temperature graph can be divided in two parts: the rapid running-in heating and the predictable temperature oscillations during stabilized friction. Due to this, using the registered data, for the heating state, a linear regression line was determined, while for the stable wear, the average temperature was calculated. The linear regression curve can be determined using the following linear equation:

(2) y=ax+b

where *a* determines the heating speed of the pin during friction. The regression lines were calculated using the Matlab software.

**Figure 6:**
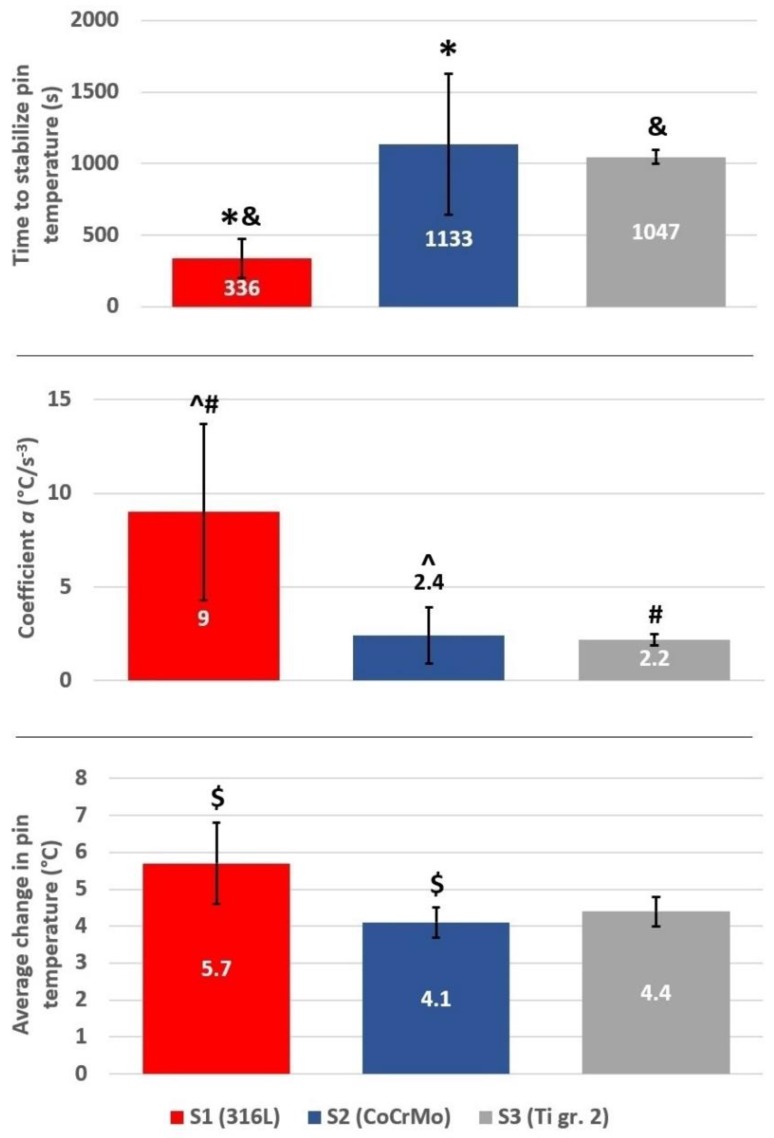
Results from the temperature measurements, mean±SD; red, 316L stainless steel; blue, CoCrMo; black, Ti gr. 2. Marks *^&^#$^ are used to denote the groups between which the statistically significant difference exists (one-way ANOVA, α = 0.05; post-hoc Tukey HSD test, α = 0.05).

Results from the temperature measurements are presented in Figure 6. Considering the limitations of the study, the material which differs the most in terms of frictional heating is the 316L steel (one-way ANOVA, α = 0.05; post-hoc Tukey HSD test, α = 0.05). Though the greatest weight loss was observed for the tribological pair 316L vs. Ti gr. 2 (Figure 4), the friction-induced heating is the greatest for the self-mated stainless steel. Moreover, the average pin temperature in the stable friction state also seems to be the highest for 316L steel. Though no statistically significant difference was found between 316L and Ti gr. 2 pins, it seems that with a greater number of samples, Ti would be characterized by a lower average pin temperature in the stable friction state.

An interesting observation is the time needed to stabilize the pin temperature during sliding (Figure 6). The material that stabilizes the fastest and, at the same time, heats up more than three times faster than the other alloys, is the 316L steel. As presented in Figure 5, in less than 6 min the self-mated 316L steel can heat up more than 4 °C from the baseline temperature. In 20 min, it will heat up more than 5 °C. However, at this point it should be stressed that the temperature of the pin was not recorded right at the friction interface, as the thermocouple was installed in the proximity to the rubbing plane. Therefore, it can be suspected that locally the temperature had risen even above 5 °C. A spark-like behavior can be seen, e.g., in 316L steel (Figure 5), where the temperature fluctuations equal up to 3 °C. Nevertheless, considering the natural body temperature which equals 37 °C, the increase in temperature at the mating interface of the rubbing surfaces by 5 °C would cause heating the implant up to 42 °C. It is an important finding as even a short friction action can cause the temperature in the implant itself and in the adjacent tissues to rise. Considering the results presented in Figure 5, one may ask a question whether the temperature increase of mating elements of 8 °C during only 7200 s (60 min) of friction might cause danger to the implant user.

In the paper by Bergmann et al. [22], it was shown that after an hour of walking, the in vivo peak temperature of a hip implant with polyethylene cup can rise to 43.1 °C. As stated in their other work, the critical temperature for soft tissue and synovial fluid is 42 °C, while the bone cells can withstand 45 °C [30]. Nevertheless, the specific heat capacity of the human tissue is approximately 7 times greater than that of biomedical metallic alloys [31,32]. Due to that, to cause a temperature rise of 1 °C in the living tissues in the proximity of an implant, an approximately 7 times greater energy in the form of heat must be transferred to the cells. Though at the first sight it does not seem very likely to happen, it is possible. First of all, during the movement, heat is generated in skeletal muscles. In a dynamic exercise, only 3 min are needed to elevate the temperature of bigger muscle groups to ca. 38 °C [33]. When adding to that high intensity repetitive sliding of the components of an endoprosthesis, critical temperatures may locally be reached.

Nowadays, the multi-component implants made of stainless still, cobalt or titanium and its alloys are used in both human and veterinary medicine in surgeries such as total knee replacement, elbow replacement, and others [3,34]. In most cases, the metallic parts are separated by polyethylene inserts [34]. Though the metal-on-metal tribological pairs are not commonly found in implants anymore, the complications resulting from wear of polyethylene liners are very well known. As presented in some papers, in both veterinary and human implants, sometimes the wear-through of the polyethylene inserts occurs [35,36], causing unintentional metal-on-metal contact of bearing surfaces in artificial joints. Moreover, as shown in the paper by Madl et al. [7], malpositioning of a MoM hip implant can result even in 100-fold increase in the yearly volumetric wear of the alloy.

The severe wear of a polyethylene insert that resulted in a metal-on-metal contact was found in a dog, only 7 years after the successful performance of total hip replacement surgery [35]. The faulty implant resulted in metallosis and formation of a pseudotumor that extended into the abdominal cavity. Interestingly, histopathology of the pseudotumor did not exhibit wear debris of an implant origin, while the tissues from the nearest implant proximity were affected by the polyethylene particles. Therefore, it might be suspected that the temperature effect might have contributed to the tissue irritation and the onset of pathological changes.

As it was presented, the analysis of the recorded COFs, the temperature alterations as well as the weight loss of three different tribological pairs does not indicate whether a simple relationship between wear, frictional heating and coefficient of friction exists. It can be said that for all analyzed materials, COF stabilized at a similar level (Figure 3). Nevertheless, at the same time it can be seen that after the short running-in state, for 316L steel, a stable, slow increase in COF is present, while in case of CoCrMo, the COF decreases after ca. 560 m of friction (Figure 2). Moreover, for Ti gr. 2 (Figure 2), much higher fluctuations of COF in time are visible. However, the average COF tends to slightly decrease in time. On the other hand, frictional heating (Figure 5 and Figure 6) is the greatest for tribological pair S1 (self-mated 316L steel), while the friction-induced weight loss is dramatically different. In terms of weight loss, the greatest wear is observed for pair S3 (Ti gr. 2 vs. 316L steel). In average, the Ti gr. 2 pin wear (Figure 4) is over 100 times greater than that of 316L, and more than 300 times greater than it was observed for CoCrMo. Therefore, in order to analyze the wear modes which caused the observed differences in the tribological behavior of discussed materials, SEM and EDX observations were done.

**Figure 7:**
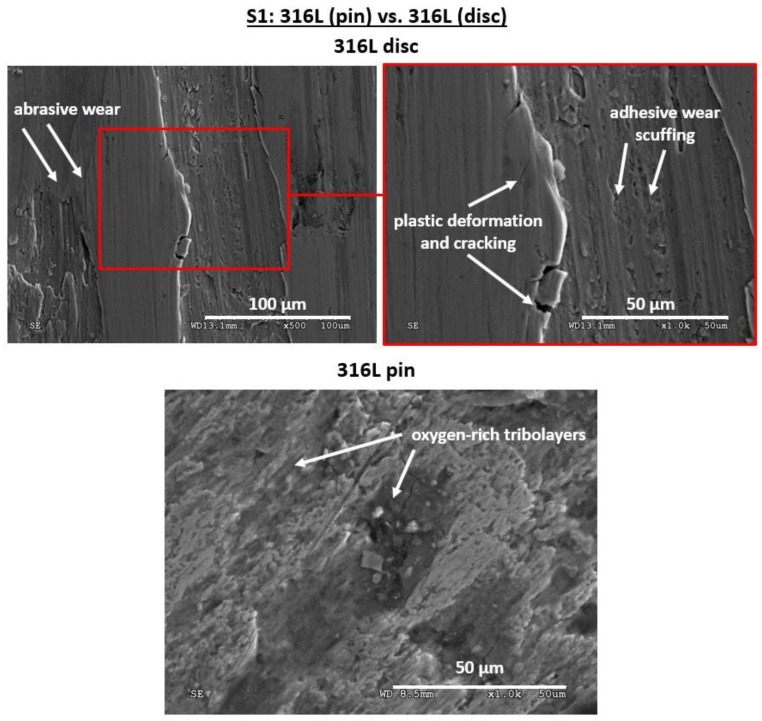
Surface morphology of the as-worn tribological pair S1: 316L pin vs. 316L disc. Signs of adhesive wear, scuffing, abrasive wear can be pin on disc, while plastic deformation and formation of oxygen-rich tribolayers took place on the pin.

For 316L stainless steel, the coefficient of friction (Figure 2) stabilizes after a short running-in state. After approx. 2000 s of sliding, until the end of the wear test, most of the time the COF fluctuates at ca. 0.4. Both the slight fluctuations and abrupt peak changes in COF in the steady wear state might be caused by the mixture of lubricating effect of transfer layers, adhesive wear of the tribological pair as well as its abrasive wear (Figure 7). Signs of scuffing between the pin and disc can be seen in Figure 7. Moreover, the material is smeared over the pin surface, as well as oxygen-rich transfer layers are present, as shown in Figure 7. The free particles of the abrasively worn steel are also seen (Figure 7). The signs of abrasive wear and microcutting are present particularly on the disc that has been mated with the pin. Nevertheless, the material smearing and its plastic deformation can also be seen in the worn track of a disc (Figure 7).

According to Hashemi et al. [37], the adhesive wear is the predominant wear mode in austenitic steels. As stated in the work by Li et al. [17], adhesive wear of austenitic stainless steels occurs when those are sliding against steels. As presented by the authors, adhesive wear causes microcracking, peeling-off the material, as well as release of wear debris which is distributed along the crack. According to Li et al. [17], at the running-in process, high contact stress occurs, thus leading to asperity fracture at the beginning of the wear test. Under such a condition, small amounts of wear particles produced via asperity fracture are crushed down between the slider and sample, leading to adhesive and abrasive wear of the tribological pair as the sliding distance increases. Similar tribological behavior of 316LVM steel was observed by Łępicka et al. [38] in the wear tests conducted in dry sliding conditions. Moreover, according to Hashemi et al. [37], when mated with AISI 52100 bearing steel, 316L also was adhesively and abrasively worn.

Similar images of surface wear were observed for CoCrMo, as presented in Figure 2. In general, it is known that hexagonal closed packed (hcp) metals are less prone to adhesive wear and scuffing than either body-centered cubic (bcc) or face-centered cubic (fcc) metals. This difference is assumed to be related with the number of operable slip systems in the crystals [39]. In the CoCrMo alloys, the fcp and the hcp crystalline structures co-exist. Though at room temperature the fcp phase is predominant, the fcp → hcp transition can be strain-induced [40]. The fcp → hcp transition of CoCrMo alloy was observed by Pourzal et al. [41] in the lubricated wear test that was conducted in a reciprocating sliding test rig. In the work by Chiba et al. [42], based on analyses of the X-ray diffraction patterns obtained for the worn tracks, it was CoCrMo alloys that are prone to strain-induced martensitic transformation (SIMT) in friction environments. Although before the tribological test, in forged CoCrMo alloy the volume fraction of fcp phase to hcp equaled 75%, after the tribological examination, mostly hcp martensite was identified in the X-ray diffraction patterns.

**Figure 8:**
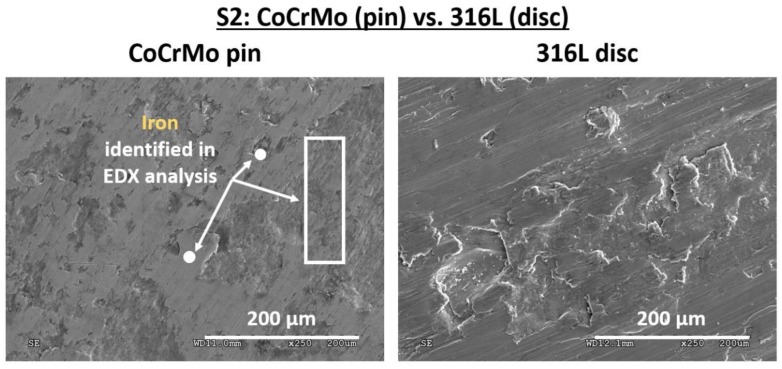
Surface morphology of the as-worn tribological pair S2: CoCrMo pin vs. 316L steel. Areas examined by EDX on the CoCrMo sample are shown in white. Material transfer from the 316L disc to the CoCrMo pin was detected, as the iron-rich transfer layers are present at the pin surface. On the other hand, plastic deformation and signs of three-body abrasive wear of stainless-steel disc can be seen.

As fcp → hcp transformation occurs in CoCrMo alloys during friction, when paired with stainless steel, CoCrMo almost does not become adhesively worn and is not transferred to the counter sample. This was confirmed in SEM-EDX analyses of the as-worn samples, as presented in Figure 8. No cobalt was transferred to the disc surface, while Fe-rich transfer layers were identified on the pin. Nevertheless, there are many microgrooves on the surface of a steel disc (Figure 8). The grooves are oriented in the sliding direction. This would imply a three-body abrasive wear of the steel surface, possibly caused by carbides originating from the CoCrMo alloy [41].

On the other hand, an entirely different tribological behavior was observed for the tribological pair stainless steel, Ti gr. 2. As presented in Figure 2, during almost the whole wear test, the coefficient of friction oscillates between 0.40 and 0.45. This behavior is typical of the slip-stick phenomena of the friction pair [43]. As the surface asperities interlock until the driving force is high enough to break the surface irregularities and slide them over one another, the relative motion does not occur until some critical shear stress is reached. In many cases, under the applied normal load, surface asperities become plastically deformed, and soon after that, micro-welding of both materials takes place [38]. When the static frictional force is greater than the kinetic frictional force during sliding, the interfaces slip each other to relieve the resultant stress [43]. While relative displacement between the pin and the disc was constantly present, the micro-welds were subsequently fractured. It is known that the slip-stick effect depends not only on the surface roughness of the mating materials, but on their elastic and plastic properties as well [43]. In this case, both titanium pin and stainless-steel counter sample suffered from severe wear. As presented in Figure 4, the greatest pin as well as counter sample weight loss was observed for the tribological pair S3, titanium vs. stainless steel (one-way ANOVA, α = 0.05; post-hoc Tukey HSD test, α = 0.05).

**Figure 9:**
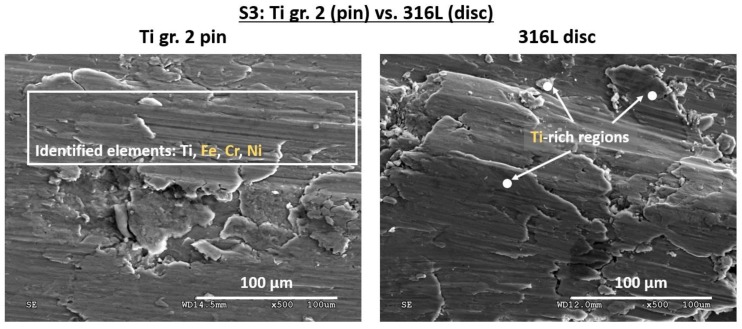
Surface morphology of the as-worn tribological pair S3: Ti pin vs. 316L steel. Areas examined by EDX are shown in white. Material transfer and similar surface damages, typical for scuffing wear, are visible on both Ti pin and 316L disc.

The slip-stick behavior of the tribological pair with Ti gr. 2 that induced severe adhesive wear of both pin and the counter sample has also been confirmed in SEM observations (Figure 9). On the titanium pin surface, numerous grooves as well as spalling-like patches are visible. Moreover, microcracked flakes are also present. According to the other authors, this type of surface damage is typical of a mixture of abrasive and adhesive wear [44]. Therefore, it can be concluded that scuffing occurred. The EDX analysis confirmed material transfer between the mating elements. On the pin surface, Fe-, Cr- and Ni-rich tribolayers were detected, while on the counter sample, high atomic concentration of Ti was revealed in the worn track.

As can be seen, the dominant wear modes of Ti gr. 2 significantly differ from the two previously discussed tribological pairs, S1 and S2. In pins made of both 316L steel and CoCrMo alloy, the SEM observations revealed that during friction, tribolayers were formed. In case of steel, the tribofilms were oxygen-rich, while for CoCrMo, the tribolayers were enriched with iron, which has been transferred from the softer steel disc. Both the oxide films on 316LVM steel, whose formation could be accelerated by the rapid temperature increase, as well as secondary layers on cobalt could protect the mating surfaces during friction. However, in case of Ti gr. 2, the transfer of materials between the mating surfaces, the plastic smearing of the transferred metals, as well as the presence of the wear particles in the friction zone may lead to formation of the new phases, including but not limited to the multi-component intermetallics, which may have an impact on further intensification of the wear processes. Despite the hardness similar to 316L steel (Table 1) and the tendency to oxidation processes, the phenomena that occur during friction of titanium are entirely different. The obtained results confirm the unfavorable frictional properties of titanium that should be taken into account in the artificial joints design processes.

Though our study has its limitations resulting from the design of the experiment, it addresses an important problem of friction-induced heating of the biomedical alloys. While the pin was worn out during the test, the change of its height and the resulting proximity of the thermocouple to the sliding plane was not taken into account. Moreover, the set sliding speed was slightly higher than the one that occurs during walking (Table 2), which could affect the temperature response of the tested materials. Nevertheless, both the heating speed during running-up and the observed average temperature in stabilized friction do not correlate with the pin wear. The greatest temperature flux was observed in 316L steel, which at the same time suffered from the slightest wear-induced material loss. On the other hand, all the tested tribological pairs differed in terms of predominant wear modes. Due to that, it can be concluded that the tendency to frictional heating is highly dependent on the mating materials. Therefore, further analyses in the field of frictional heating of biomedical materials are recommended.

## 4. Conclusions

In this paper, a frictional behavior of 316L stainless steel, CoCrMo cobalt alloy as well as Ti gr. 2 titanium was tested in a tribological pair with stainless steel. During the test, both changes in the friction force as well as in the pin temperature were registered. According to the results provided, the following conclusions can be drawn:

1. When sliding in the environment of a saline solution, the tested materials significantly differ in terms of the observed wear modes and the intensity of the wear phenomena. The greatest wear was observed for the tribological pair Ti gr. 2 vs. 316L stainless steel, where the predominant wear mode was the adhesive wear and scuffing-like behavior. The oscillations of the friction force registered during the sliding test might be caused by the slip-stick behavior of the both materials: micro welding on the surface asperities and the subsequent tearing off the material particles.
2. As presented in the study, the temperature behavior of biomedical alloys is dependent on the tribological pair. During the sliding test, in the self-mated 316L steel, only 5 min of friction were enough to cause the temperature to rise inside the pin 5.7 °C from the baseline. Therefore, it can be suspected that sliding of the mating parts of an endoprosthesis, e.g., during a vigorous walk, can cause the temperature to locally rise, especially in the bearing surfaces. Taking into account the critical temperature for tissues and physiological fluids which equals 42 °C, the temperature changes can cause irreversible changes in the lubricating fluids of an artificial joint.

## Figures and Tables

**Table 1:** Summary of all the tribological pairs tested.

Frictional Pair No.	Pin Material	Disc Material	Pin Ra (µm)	Vickers Microhardness of the Pin Material (HV_0.1_)
*S1*	316L	316L	0.015 ± 0.001	195 [23]
*S2*	CoCrMo	316L	0.014 ± 0.001	440 [24]
*S3*	Ti gr. 2	316L	0.022 ± 0.002	190 [25]

**Table 2:** The in vivo sliding speed of a hip implant measured during walking [26].

Age (Years)	Gender	Mean Sliding Speed (m/s)	
		Extension	Flexion
56	m	0.02	0.04
62	m	0.03	0.05
60	m	0.02	0.06
50	m	0.03	0.06
62	f	0.02	0.08
69	m	0.03	0.05
53	m	0.03	0.06
56	m	0.03	0.06
